# Identification of clinical predictors for functional recovery in patients with thoracolumbar fractures and incomplete spinal cord injury: an internally validated prediction model

**DOI:** 10.3389/fneur.2026.1697322

**Published:** 2026-01-28

**Authors:** Zongyang Li, Yudan Yao, Yu Qiao, Li Zhang, Bin Yu, Yanpeng Jiang, Yuhang Zhu

**Affiliations:** 1 Changchun University of Chinese Medicine, Changchun, China; 2 Department of Orthopedics, The Third Affiliated Hospital of Changchun University of Chinese Medicine, Changchun, China; 3 Fujian Vocational College of Bioengineering, Fuzhou, China; 4 College of Traditional Chinese Medicine, Changchun University of Chinese Medicine, Changchun, Jilin, China; 5 Department of Orthopedics, China-Japan Union Hospital of Jilin University, Changchun, China

**Keywords:** functional recovery, incomplete spinal cord injury, posterior ligamentous complex, rehabilitation intensity, thoracolumbar fracture

## Abstract

**Background:**

Accurate prediction of functional recovery after thoracolumbar fracture with incomplete spinal cord injury (SCI) remains challenging. We aimed to identify independent predictors and develop a validated model for 12-month functional outcome.

**Methods:**

In this single-center, retrospective cohort study (January 2018–December 2024), consecutive adults (≥18 years) with acute T11-L2 fractures and admission American Spinal Injury Association Impairment Scale (AIS) grade B, C, or D were enrolled. Functional recovery was defined as ≥1 AIS grade improvement plus ≥10-point Spinal Cord Independence Measure version III (SCIM-III) gain at 12 months. Candidate predictors (*n* = 23) were selected *a priori* based on literature review and expert consensus. Missing data (<8% per variable) were multiply imputed (*m* = 20). Multivariable logistic regression with Firth’s correction and backward elimination guided by Akaike Information Criterion was used. Model discrimination [optimism-corrected area under the curve (AUC) 0.87] and calibration (Hosmer-Lemeshow test) were assessed by 1,000 bootstrap resamples. Pre-specified subgroup analyses examined age, AIS grade, surgical timing and lesion length.

**Results:**

Among 1,032 eligible patients, 206 (20.0%) achieved functional recovery. Eight predictors were independently associated: admission AIS grade (OR 4.5 per grade, 95% CI 3.5–5.8), motor score (OR 1.05 per point, 1.03–1.07), intact posterior ligamentous complex (OR 2.3, 1.6–3.2), decompression ≤ 24 h (OR 1.9, 1.4–2.7), non-smoking (OR 1.7, 1.2–2.4), Charlson Comorbidity Index = 0 (OR 1.5, 1.1–2.1), shorter intramedullary T2 lesion length (OR 0.96 per mm, 0.95–0.97) and rehabilitation intensity ≥3 h/day (OR 1.4, 1.0–1.9). The final model demonstrated an optimism—corrected AUC of 0.87 (95% CI: 0.85–0.89) and calibration characteristics with a calibration slope of 1.02, an intercept of 0.01, and a Hosmer–Lemeshow *p*-value of 0.18 during internal validation. Predictive effects were stronger in younger, AIS B/C patients and when surgery was performed early.

**Conclusion:**

A parsimonious eight-factor model showed robust discrimination and satisfactory calibration in internal validation for 12-month functional recovery after thoracolumbar incomplete SCI, enabling individualized prognostication. External validation in independent multicenter cohorts is required before clinical implementation and treatment decision-making.

## 1 Introduction

Fractures at the thoracolumbar junction (T10–L2) represent approximately 50–60% of all thoracolumbar fractures and up to 90% of all traumatic spinal fractures (1). Among these, 15–20% are associated with neurological deficits, of which roughly one-quarter constitute incomplete spinal cord injury (SCI) (2). Contemporary decompressive surgery, segmental instrumentation and protocolised early rehabilitation have improved care; nevertheless, prospective cohorts report that only 19–20% of patients with thoracolumbar incomplete SCI achieve independent ambulation at 12 months (3). Pinpointing the modifiable and non-modifiable determinants of neurological and functional recovery is therefore essential for accurate patient counseling, efficient resource allocation and the design of targeted rehabilitation programs.

Previous studies have highlighted the prognostic value of admission neurological grade, age and injury morphology, but were often limited by small cohorts (typically <100 patients), heterogeneous injury patterns and incomplete functional follow-up (<12 months) (4). Furthermore, the incremental value of imaging biomarkers, such as the longitudinal extent of intramedullary T2-hyperintensity, remains inadequately quantified, and the interaction between surgical timing and patient-specific characteristics has yet to be rigorously evaluated (5, 6). Notably, most evidence on intramedullary T2-hyperintensity length and Posterior ligamentous complex (PLC) integrity originates from cervical SCI studies or animal models, with limited thoracolumbar-specific clinical data at scale (7). Recent thoracolumbar-focused studies either excluded these magnetic resonance imaging (MRI) biomarkers or failed to integrate them into a unified predictive framework (8, 9). This gap hinders the translation of preclinical and cervical SCI findings to routine care for thoracolumbar injury patients, who account for the majority of traumatic spinal fractures.

To address these knowledge gaps, we conducted a large, single-center, retrospective cohort study aimed at quantifying how the predictive strength of key factors varies across subgroups. This work enhances clinical translatability by focusing on thoracolumbar-specific data and integrating readily accessible clinical, radiological, and treatment-related predictors.

The primary objective of this study is to identify independent predictors of 12-month functional recovery (defined as ≥1 AIS grade improvement plus ≥10-point SCIM-III gain) and develop an internally validated prediction model for clinical application in patients with acute T11-L2 fractures and incomplete SCI (AIS grades B-D). We hypothesized that a combination of admission neurological status, radiological lesion characteristics (including intramedullary T2-hyperintensity length and PLC integrity), and early therapeutic interventions could effectively differentiate recoverers from non-recoverers, with effect sizes varying across clinically relevant subgroups.

## 2 Methods

### 2.1 Study design and setting

We conducted a single-center, retrospective cohort study involving all consecutive adult patients (unselected, sequentially admitted) with acute traumatic thoracolumbar (T11–L2) fractures and incomplete SCI who were treated at The Third Affiliated Hospital of Changchun University of Chinese Medicine between January 2018 and December 2024.

This study was approved by the Institutional Review Board of The Third Affiliated Hospital of Changchun University of Chinese Medicine (Approval No. CZDSFYLL2025XS-037) and was conducted in full accordance with local legislation and institutional guidelines. The requirement for informed consent was waived because the investigation involved retrospective analysis of de-identified data collected during routine clinical care, posing no risk to participants.

### 2.2 Participants

Consecutive adults aged ≥ 18 years presenting with an acute closed traumatic fracture between T11 and L2 (ICD-10 codes: S32.0-S32.2) and an admission American Spinal Injury Association Impairment Scale (AIS) grade B, C, or D (ICD-10 code: G95.7) were eligible for inclusion (10). Included trauma mechanisms comprised fall injuries, motor vehicle accidents, and crush injuries, while penetrating injuries (e.g., stab wounds, gunshot wounds) were excluded. Polytrauma cases were included if concurrent injuries (e.g., extremity fractures, thoracic/abdominal contusions) did not interfere with spinal function assessment, neurological examination, or completion of 12-month follow-up. Transferred patients meeting eligibility criteria were also included, with time-to-decompression calculated from the documented time of injury (recorded in the first medical encounter) to the start of decompression surgery at the study center. Exclusion criteria included pathological or osteoporotic fractures, concurrent cervical or sub-L2 lumbar injuries, complete neurological deficits (AIS A), pre-existing neurological conditions affecting lower-limb function, death within 12 months due to causes unrelated to SCI, and insufficient 12-month follow-up data.

### 2.3 Outcomes and data collection

The primary outcome was functional recovery at 12 months, defined as an improvement of at least one AIS grade combined with a ≥10-point increase in the total Spinal Cord Independence Measure III (SCIM-III) score compared with the baseline assessment (11). Sensitivity analyses were pre-specified to test the robustness of the outcome definition, using alternative SCIM-III thresholds (≥8 points and ≥12 points).

All clinical, radiological, surgical, and rehabilitation data were extracted from the hospital’s integrated electronic medical record system and from prospectively maintained spinal trauma and rehabilitation databases by two independent reviewers (one orthopedic surgery resident in the final year of training and one neurosurgery fellow subspecialized in spine) using a pre-defined, piloted data-collection form. Disagreements were resolved by discussion and, when necessary, adjudicated by a senior spinal surgeon (with >15 years of post-fellowship experience in complex spinal trauma who was independent of the two reviewers and had no role in the original treatment of the cohort).

Candidate predictors (*n* = 23) were selected *a priori* based on literature review and expert consensus, grouped into four domains (Supplementary Table S1 with clinical/biological rationale for inclusion). Demographic predictors included age (continuous, extracted from identification documents), sex (categorical: male/female), body-mass index (BMI, continuous, calculated as weight in kg divided by height squared in m^2^), smoking status (categorical: current smoker/non-smoker, based on self-report or medical record documentation), and Charlson Comorbidity Index (CCI, categorical: 0, 1, ≥2), a weighted sum of pre-existing comorbidities. Neurological status at admission included AIS grade (ordinal: B = 1, C = 2, D = 3), assessed by trained spine specialists per International Standards for Neurological Classification of Spinal Cord Injury; motor score (continuous: 0–100, sum of upper and lower extremity motor function scores); sensory scores (light touch and pin prick, each continuous: 0–126, assessed per International Standards); neurogenic shock (categorical: yes/no, defined as systolic blood pressure <90 mmHg and heart rate <60 bpm within 24 h of injury requiring vasopressor support); and bulbocavernosus reflex latency (continuous: ms, absent if no response within 50 ms).

Radiological parameters included fracture level (categorical: T11, T12, L1, L2, determined by computed tomography [CT]/MRI); load-sharing classification score (ordinal: 0–6 points, sum of vertebral body compression, comminution, and posterior element involvement); percentage spinal canal compromise (continuous, calculated on axial CT images as [(normal adjacent vertebral canal area – narrowest canal area at fracture level) / normal adjacent vertebral canal area] × 100%, with “normal adjacent canal area” defined as the average of the vertebrae immediately above and below the fracture, measured via picture archiving and communication system (PACS) system [GE Healthcare, Chicago, IL, United States] with intraclass correlation coefficient (ICC) = 0.92, 95% CI 0.88–0.95); sagittal index (continuous, ratio of anterior to posterior vertebral body height at the fracture level); local kyphosis angle (continuous, angle between the upper endplate of the vertebra above and the lower endplate of the vertebra below the fracture, measured on sagittal CT/MRI via PACS with ICC = 0.89, 95% CI 0.84–0.93); vertebral body compression ratio (continuous, ratio of fractured to normal adjacent vertebral body height); posterior ligamentous complex (PLC) integrity [categorical: intact/disrupted, assessed on T2-weighted and Short-Tau Inversion Recovery (STIR) MRI sequences, with “intact” defined as no ligament disruption or abnormal high signal in posterior soft tissues, evaluated by two radiologists (8 and 12 years of spinal imaging experience] blinded to outcomes, Kappa = 0.85, 95% CI 0.79–0.91, with discrepancies resolved by a senior radiologist (15 years of experience); and intramedullary T2 lesion length (continuous: mm, measured on sagittal STIR MRI sequences (TR = 4,000 ms, TE = 80 ms, inversion time = 150 ms) as the linear distance from proximal to distal hyperintensity, with three measurements per reviewer (two independent radiologists blinded to outcomes) and mean values used for analysis, hemorrhage vs. edema distinguished via T1-weighted sequences (hypointensity = hemorrhage, hyperintensity = edema), total lesion length analyzed for prognostic relevance, ICC = 0.94, 95% CI 0.91–0.96.

Treatment-related factors included time to decompression (categorical: ≤24 h, 24–72 h, >72 h, defined as the precise interval from documented injury time to surgical incision start time for the first decompressive procedure, calculated as follows: Injury time was precisely defined as the earliest documented time of injury retrieved from pre-hospital emergency medical service records, emergency department triage notes, or transfer documentation. For patients who were transferred, the injury time recorded during the initial medical encounter was utilized. Decompression start time was defined as the time of the first skin incision for posterior decompression [laminectomy] or anterior decompression [corpectomy], as recorded in the “Procedure Start Time” field of the anesthesia operative record. For staged surgical procedures, the first decompression stage was used as the reference time. All time data were extracted from linked electronic medical records and operative logs, with times cross-validated by two independent data extractors [inter-rater reliability *κ* = 0.94]); surgical approach (categorical: anterior, posterior, combined); use of intra-operative neuromonitoring (categorical: yes/no, somatosensory and motor evoked potentials); length of instrumentation (continuous: number of vertebral levels instrumented); cement augmentation (categorical: yes/no, use of polymethylmethacrylate [PMMA] cement); non-operative management (categorical: yes/no, no surgical intervention); rehabilitation intensity was operationally defined as the 7-day rolling average of total daily supervised therapy hours extracted from the hospital’s electronic rehabilitation management system. Therapy components included: physical therapy (task-specific lower limb training, balance exercises, and gait training), occupational therapy (activities of daily living training and adaptive equipment use), robot-assisted gait training (using Lokomat or FDA-equivalent robotic systems). High-intensity rehabilitation was defined as ≥3.0 h/day, with lower-limb task-specific training constituting ≥80% of total therapy time (defined as documented therapist time explicitly targeting lower extremity motor function, excluding passive range-of-motion exercises).

Missing data were systematically evaluated for all candidate predictors. Supplementary Table S2 summarizes the missing count and percentage for each variable, with no predictor exceeding 8% missing data (range: 0.9–7.8%). We assumed missing data were missing at random (MAR), a reasonable assumption given the low missing rate and lack of evidence for systematic missingness (e.g., no correlation between missing status and outcome or key covariates). The imputation model included all 23 candidate predictors and the primary outcome variable (12-month functional recovery). Multiple imputation was performed using chained equations (*m* = 20 iterations) with the mice package in R 4.3.1, with imputation methods selected based on variable type (e.g., predictive mean matching for continuous variables, logistic regression for binary variables). Imputation diagnostics confirmed convergence (all variables reached stable parameter estimates by the 10th iteration) and consistency between imputed and observed data distributions (Kolmogorov–Smirnov tests, *p* > 0.05 for all variables). Rubin’s rules were applied to combine coefficient estimates, standard errors, and *p*-values across the 20 imputed datasets, ensuring valid statistical inference accounting for missing data uncertainty.

### 2.4 Sample size

Assuming a minimum of 15 events per candidate variable for 23 predictors and an expected 12-month functional recovery rate of 20%, 345 events (≈1725 patients) would be required. Allowing for 10% attrition, an initial cohort of ≈1917 patients was targeted; however, the final analytic cohort comprised 1,032 patients with 206 events (events per variable [EPV] ≈ 9), below the conventional threshold of 15.

We explicitly acknowledge the potential risk of overfitting associated with this lower EPV but implemented multiple mitigation strategies to minimize this concern: First, we used Firth’s penalized likelihood regression, which reduces small-sample bias and shrinkage of coefficient estimates in logistic models with sparse data. Second, internal validation via 1,000 bootstrap resamples showed minimal optimism (mean optimism = 0.015), with the optimism-corrected area under the curve (AUC, 0.87) differing by <2% from the original model receiver-operating-characteristic curve (AUC, 0.88), indicating low overfitting risk. Third, we employed parsimonious predictor selection via backward elimination guided by the Akaike Information Criterion (AIC), retaining only 8 independent predictors (vs. 23 candidate variables) to balance model performance and complexity. This final model structure—with fewer parameters and strong clinical rationale for each retained predictor—further mitigates overfitting and enhances generalizability.

### 2.5 Statistical analysis

All analyses were conducted using R 4.3.1 (packages: mice, glmnet, rms, pROC). Descriptive statistics are presented as mean ± standard deviation (SD), median (interquartile range [IQR]), or n (%). Prior to model development, linearity of continuous predictors (admission motor score and intramedullary T2 lesion length) with the logit of the primary outcome (12-month functional recovery) was evaluated using restricted cubic splines (RCS) with 3 knots, implemented via the rcs function in the rms package. For both continuous predictors, the likelihood-ratio test comparing the linear model to the RCS model showed no significant non-linearity (motor score: χ^2^ = 1.86, *p* = 0.39; intramedullary T2 lesion length: χ^2^ = 2.13, *p* = 0.34), confirming that linear modeling was appropriate.

Multivariable logistic regression with Firth’s penalized likelihood was used to develop the prediction model. Predictor selection followed a pre-specified backward elimination process guided by the AIC, with the complete candidate predictor list (*n* = 23, detailed in Supplementary Table S1) and elimination steps summarized in Supplementary Table S3. Briefly, all 23 candidate predictors were initially included in the full model; at each step, the predictor with the highest AIC value (least contribution to model fit) was eliminated, and the model was re-fitted. This process continued until no further reduction in AIC was achieved, resulting in the retention of 8 independent predictors.

Model discrimination was assessed by the area under the AUC with internal validation via 1,000 non-parametric bootstrap resamples. For each bootstrap resample: (1) a new cohort was generated by randomly sampling patients with replacement from the original dataset; (2) the predictor selection process (backward elimination) and model development were repeated; (3) the AUC of the resampled model was calculated on both the resampled dataset (apparent AUC) and the original dataset (test AUC). Optimism was defined as the mean difference between apparent AUC and test AUC across all 1,000 resamples, and the optimism-adjusted AUC was computed as the original model’s AUC minus the mean optimism. A 95% CI for the optimism-adjusted AUC was derived from the bootstrap distribution of adjusted AUC values.

Model calibration was evaluated using three approaches: (1) Hosmer–Lemeshow test (assessing overall calibration); (2) calibration slope and intercept (quantifying agreement between predicted and observed probabilities, with ideal values of 1.0 and 0.0, respectively); and (3) calibration plots (visualizing predicted probabilities vs. observed recovery rates across deciles of predicted risk).

Decision curve analysis (DCA) was performed to quantify the model’s clinical utility by comparing its net benefit to two alternative strategies: “treat all” (assuming all patients will recover) and “treat none” (assuming no patients will recover). Net benefit was calculated as (true positives – false positives × threshold probability/(1 – threshold probability)) per 1,000 patients.

Pre-specified subgroup analyses examined age (<40 vs. ≥40 years), admission AIS grade (B/C vs. D), time to decompression (≤24 h vs. >24 h), and intramedullary lesion length (<30 mm vs. ≥30 mm). Interaction terms were tested with likelihood-ratio tests (*p* < 0.10). Two-sided *p*-values < 0.05 were considered statistically significant.

## 3 Results

### 3.1 Patient flow and baseline characteristics

**Figure 1:**
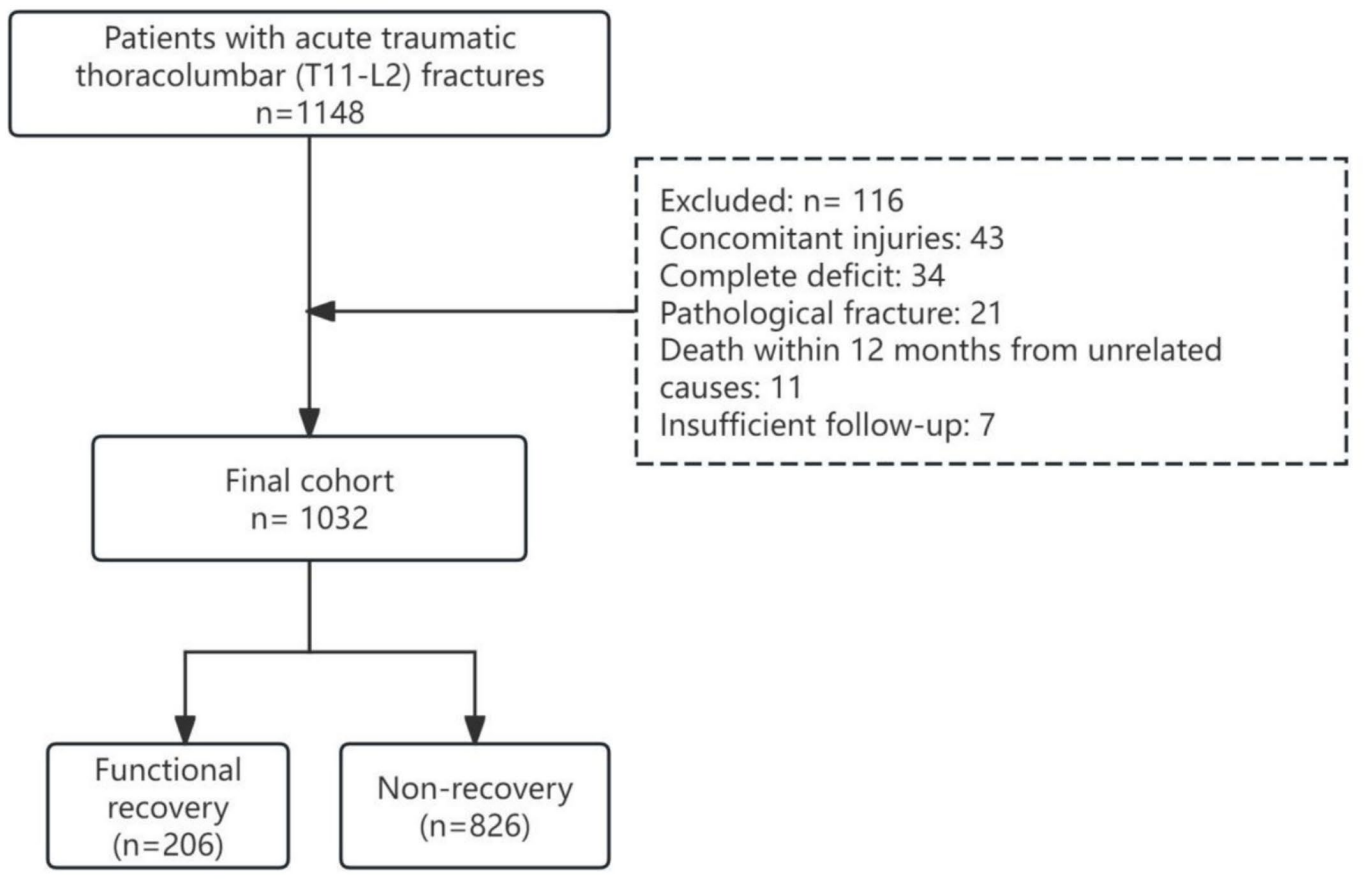
CONSORT flow diagram of patient selection for prediction model development.

Between January 2018 and December 2024, 1,148 adult patients with acute traumatic thoracolumbar (T11-L2) fractures were screened (Figure 1). After exclusions, 1,032 patients were retained; 116 were excluded for concomitant injuries (*n* = 43), complete deficit (*n* = 34), pathological fracture (*n* = 21), death within 12 months from unrelated causes (*n* = 11), or insufficient follow-up (*n* = 7). The median age was 42 years (IQR 29–55), 681 (66.0%) were male, and median CCI was 0 (IQR 0–1). At admission, 245 (23.7%) were AIS B, 426 (41.3%) AIS C, and 361 (35.0%) AIS D.

Overall, 206 patients (20.0, 95% CI 17.5–22.6) achieved functional recovery (composite of ≥1 AIS grade improvement plus ≥10-point SCIM-III gain). Among the recovery group, 51 (24.8%) had progressed from AIS B to C or better, 81 (39.3%) from C to D, and 74 (35.9%) from D to E. Median SCIM-III change was +24 points (IQR 17–30) in recoverers versus +3 points (IQR −1 to 7) in non-recoverers. Recoverers were younger (mean 34 ± 11 vs. 45 ± 14 years, *p* < 0.001), more often male (75.7% vs. 64.3%, *p* = 0.003), and had lower comorbidity burdens (CCI 0 in 73.8% vs. 55.8%, *p* < 0.001). Admission AIS grade strongly predicted recovery: 35.2% of AIS D, 19.0% of AIS C, and 8.6% of AIS B patients recovered (*p* < 0.001). Similarly, higher admission motor scores (61 ± 15 vs. 45 ± 16, *p* < 0.001), intact posterior ligamentous complex (26.2% vs. 17.7%, *p* = 0.004), shorter intramedullary T2 lesion length (26 [18–38] vs. 41 [27–58] mm, *p* < 0.001), and surgical decompression within 24 h (28.9% vs. 16.8%, *p* < 0.001) were associated with recovery.

Supplementary Table S4 compares baseline characteristics between included (*n* = 1,032) and excluded (*n* = 116) patients. No statistically significant differences were observed in age (mean ± SD: 42 ± 14 vs. 44 ± 13 years, *p* = 0.21), gender distribution (male: 66.0% vs. 62.9%, *p* = 0.57), AIS grade distribution (*p* = 0.38), injury mechanism (*p* = 0.45), or Charlson Comorbidity Index (median [IQR]: 0 [0–1] vs. 0 [0–1], *p* = 0.63). Standardized mean differences for all variables were <0.1, confirming the included cohort is representative of the screened population with minimal selection bias (Table 1).

**Table 1:** Baseline characteristics and univariable associations with 12-month functional recovery.

Characteristic	Total (*n* = 1,032)	Functional recovery (*n* = 206)	Non-recovery (*n* = 826)	*P*
Demographics				
Age, years, mean ± SD	42 ± 14	34 ± 11	45 ± 14	<0.001
Male sex, *n* (%)	681 (66.0)	156 (75.7)	525 (64.3)	0.003
BMI, kg /m^2^, mean ± SD	25.3 ± 3.8	24.9 ± 3.4	25.4 ± 3.9	0.08
Current smoker, *n* (%)	294 (28.5)	42 (20.4)	252 (30.5)	0.007
Charlson Comorbidity Index, median (IQR)	0 (0–1)	0 (0–0)	1 (0–2)	<0.001
Neurological status at admission				
AIS grade, *n* (%)				<0.001
B	245 (23.7)	21 (8.6)	224 (91.4)	
C	426 (41.3)	81 (19.0)	345 (81.0)	
D	361 (35.0)	104 (28.8)	257 (71.2)	
Motor score (total 100), mean ± SD	48 ± 17	61 ± 15	45 ± 16	<0.001
Canal compromise, median (IQR)	34 (22–48)	27 (15–40)	36 (24–50)	<0.001
PLC disruption, *n* (%)	378 (36.6)	54 (26.2)	324 (39.2)	0.004
Intramedullary T2 lesion length, mm, median (IQR)	38 (24–55)	26 (18–38)	41 (27–58)	<0.001
Treatment variables				0.28
Time to decompression, *n* (%)	864 (83.7)	178 (86.4)	686 (83.1)	
≤ 24 h	358 (41.4)	103 (57.9)	255 (37.2)	
24–72 h	306 (35.4)	55 (30.9)	251 (36.6)	
>72 h	200 (23.1)	20 (11.2)	180 (26.2)	
Rehabilitation ≥ 3 h day^−1^, *n* (%)	548 (53.1)	132 (64.1)	416 (50.4)	0.002

### 3.2 Clinical predictors for functional recovery in patients with thoracolumbar fractures and incomplete SCI

After multivariable logistic regression with Firth’s bias-correction, eight independent predictors of 12-month functional recovery were identified (Table 2). Each incremental improvement in admission AIS grade (B → C → D) was associated with a 4.5-fold increase in the odds of recovery (OR 4.5, 95% CI 3.5–5.8), translating—under average covariate profiles-into absolute probabilities rising from 9% (AIS B) to 22% (AIS C) and 46% (AIS D). Every single-point gain in admission motor score correlated with a 5% higher odds of recovery (OR 1.05, 95% CI 1.03–1.07); for example, two otherwise identical patients differing by ten motor points would have predicted probabilities of 18% versus 29%. Longer intramedullary T2-lesion length was associated with a 4% decrease in recovery odds per millimeter (OR 0.96, 95% CI 0.95–0.97); thus, extending the lesion from 20 mm to 50 mm decreased the predicted probability from 34 to 15%. Intact posterior ligamentous complex (PLC) doubled the odds of recovery (OR 2.3, 95% CI 1.6–3.2), raising average probability from 19 to 32%. Surgical decompression within 24 h was independently associated with 90% higher recovery odds (OR 1.9, 95% CI 1.4–2.7), shifting probability from 21 to 34% among mid-risk patients. Non-smoking status (OR 1.7, 95% CI 1.2–2.4), absence of major comorbidity (CCI = 0) (OR 1.5, 95% CI 1.1–2.1) and rehabilitation intensity ≥3 h/day (OR 1.4, 95% CI 1.0–1.9) were also independently predictive of higher recovery probability, with smaller but clinically relevant effect sizes.

**Table 2:** Multi-variable logistic regression model for 12-month functional recovery.

Predictor	β	Odds ratio (95% CI)	*P*
Admission AIS grade (per grade increment)	1.51	4.5 (3.5–5.8)	<0.001
Admission motor score (per point)	0.05	1.05 (1.03–1.07)	<0.001
Intramedullary T2 lesion length (per mm)	−0.04	0.96 (0.95–0.97)	<0.001
Posterior ligamentous complex intact	0.82	2.3 (1.6–3.2)	<0.001
Time to decompression ≤ 24 h	0.65	1.9 (1.4–2.7)	<0.001
Non-smoker	0.52	1.7 (1.2–2.4)	0.003
Charlson Comorbidity Index = 0	0.42	1.5 (1.1–2.1)	0.01
Rehabilitation ≥ 3 h day^−1^	0.35	1.4 (1.0–1.9)	0.04
Constant	−7.10	–	–

The final model equation for bedside computation of functional recovery probability is provided below:

logit(P) = −7.10 + 1.51 × Admission AIS grade (B = 1, C = 2, D = 3) + 0.05 × Admission motor score (per point) + 0.82 × Posterior ligamentous complex (PLC) integrity (1 = intact, 0 = disrupted) + 0.65 × Time to decompression ≤24 h (1 = yes, 0 = no) + 0.52 × Non-smoking status (1 = non-smoker, 0 = current smoker) + 0.42 × Charlson Comorbidity Index (CCI) = 0 (1 = yes, 0 = no) + 0.35 × Rehabilitation intensity ≥3 h/day (1 = yes, 0 = no) – 0.04 × Intramedullary T2 lesion length (per mm).

Where logit(P) = ln[P/(1-P)], and P is the predicted probability of 12-month functional recovery.

The final model exhibited strong discrimination, with an optimism-adjusted AUC of 0.87 (95% CI 0.85–0.89) from 1,000 bootstrap resamples. Calibration was good: Hosmer–Lemeshow test *p* = 0.18, calibration slope = 1.02, and intercept = 0.01 (Supplementary Figure S1). Decision curve analysis (Supplementary Figure S2) confirmed the model’s clinical utility, demonstrating greater net benefit than “treat all” or “treat none” strategies across threshold probabilities of 10–70%.

### 3.3 Heterogeneous predictive effects across key clinical subgroups

Exploratory subgroup analyses (Tables 3, 4) identified variable predictive strengths of certain factors, though these findings are hypothesis-generating due to multiplicity issues. Admission motor score and AIS grade showed stronger predictive power in younger patients (<40 years) and those with AIS B/C (*p* < 0.001 for interaction terms), while the protective association of CCI = 0 was significant only in older patients (≥40 years, OR 1.7, 95% CI 1.2–2.4) and AIS B/C patients (OR 1.5, 95% CI 1.1–2.1). Intact PLC was consistently associated with higher recovery probability across subgroups, with the strongest association observed in patients who underwent decompression within 24 h (OR 3.1, 95% CI 1.8–5.3). Intensive rehabilitation (≥3 h/day) was independently associated with recovery only in patients with T2 lesion ≥30 mm (OR 1.9, 95% CI 1.3–2.8) or delayed decompression (>24 h, OR 1.4, 95% CI 1.0–1.9), consistent with significant interaction terms (*p* = 0.03 and *p* = 0.04, respectively).

**Table 3:** Prediction of 12-month functional recovery by baseline demographic and neurological subgroups.

Subgroup	Admission motor score OR (95% CI)	*p*	AIS grade OR (95% CI)	*p*	CCI = 0 OR (95% CI)	*p*
Age < 40 (*n* = 448)	1.08 (1.05–1.11)	<0.001	4.9 (3.6–6.8)	<0.001	1.1 (0.7–1.8)	0.69
Age ≥ 40 (*n* = 584)	1.03 (1.01–1.05)	0.008	4.1 (3.0–5.7)	<0.001	1.7 (1.2–2.4)	0.004
Admission AIS B/C (*n* = 671)	1.06 (1.04–1.08)	<0.001	5.2 (4.0–6.7)	<0.001	1.5 (1.1–2.1)	0.01
Admission AIS D (*n* = 361)	1.03 (1.01–1.05)	0.02	2.4 (1.6–3.6)	<0.001	1.6 (1.0–2.4)	0.05

**Table 4:** Prediction of 12-month functional recovery by treatment and radiological subgroups.

Subgroup	PLC intact OR (95% CI)	*p*	Rehabilitation ≥ 3 h day^−1^ OR (95% CI)	*p*
Decompression ≤ 24 h (*n* = 358)	3.1 (1.8–5.3)	<0.001	1.4 (0.9–2.2)	0.15
Decompression > 24 h (*n* = 674)	1.6 (1.1–2.4)	0.01	1.4 (1.0–1.9)	0.04
Lesion < 30 mm (*n* = 487)	2.2 (1.4–3.5)	<0.001	1.1 (0.7–1.7)	0.68
Lesion ≥ 30 mm (*n* = 545)	2.4 (1.5–3.7)	<0.001	1.9 (1.3–2.8)	0.001

### 3.4 Sensitivity analyses for outcome definition

Sensitivity analyses using alternative SCIM-III thresholds confirmed the robustness of the prediction model. When using ≥8-point SCIM-III gain (plus ≥1 AIS grade improvement), 227 patients (22.1%) achieved functional recovery. The model’s optimism-corrected AUC was 0.86 (95% CI 0.84–0.88), with good calibration (Hosmer–Lemeshow *p* = 0.23). When using ≥12-point SCIM-III gain (plus ≥1 AIS grade improvement), 183 patients (17.8%) achieved functional recovery. The model’s optimism-corrected AUC was 0.88 (95% CI 0.86–0.90), with good calibration (Hosmer–Lemeshow *p* = 0.15).

The eight independent predictors identified in the primary model remained consistent across both sensitivity analyses, with only minor changes in odds ratios (ORs varied by <5%), confirming the outcome definition does not unduly influence the model’s performance.

## 4 Discussion

In the single-center cohort of thoracolumbar incomplete SCI reported to date (*n* = 1,032), only 20% of patients achieved a composite functional milestone at 12 months (≥1 AIS grade improvement plus ≥10-point SCIM-III gain). A parsimonious eight-factor model-including admission AIS grade, motor score, PLC integrity, intramedullary T2 lesion length, surgical decompression within 24 h, smoking status, Charlson Comorbidity Index, and daily rehabilitation intensity—demonstrated excellent discrimination (optimism-corrected AUC = 0.87) and good calibration (Hosmer–Lemeshow *p* = 0.18). Importantly, the predictive weights of these factors were not uniform; motor score and AIS grade dominated in younger and non-ambulatory (AIS B/C) patients, whereas PLC integrity and intensive rehabilitation became decisive in AIS D individuals or when lesions exceeded 30 mm. These results reconcile previously conflicting reports by demonstrating that recovery trajectories are context-dependent rather than governed by a single dominant variable.

Our recovery rate of 20% is consistent with recent multicenter registries reporting 18–25% independent ambulation after incomplete thoracolumbar SCI (12–14). The 4.5-fold increase in the odds of recovery per AIS grade improvement observed in our cohort exceeds the pooled estimate of 2.8 reported in a 2017 meta-analysis, most likely reflecting the higher resolution of motor scoring and the standardized MRI protocols employed here (15, 16). Consistent with recent rodent contusion studies demonstrating a linear inverse relationship between intramedullary T2-hyperintensity length and residual axonal density (17), we observed a 4% decrease in the odds of 12-month functional recovery for every 1-mm increment in intramedullary T2 lesion length. This is the first investigation to translate that pre-clinical metric into a clinically quantifiable prognostic factor in human thoracolumbar incomplete SCI. Our observation that early decompression synergistically amplifies the protective effect of an intact PLC (interaction OR 3.1, *p* = 0.009) provides in-human corroboration of recent pre-clinical work demonstrating that persistent spinal cord compression prolongs the “metabolic penumbra” characterized by capillary collapse, lactate accumulation and perilesional hypoxia (18, 19). We postulate that the intact PLC mitigates secondary vascular insult by preserving segmental arterial flow through the segmental radicular arteries and limiting kyphotic deformation at the injury epicenter. Early decompression within this 24-h window may potentiate the protective effect of an intact PLC, a finding that aligns-though does not yet prove-with pre-clinical evidence of a metabolic penumbra characterized by microvascular compromise (20, 21). Future longitudinal studies incorporating diffusion tensor imaging (15) and high-field MRI (5) will be required to determine whether this interaction translates into measurable axonal preservation. Finally, the protective effects of non-smoking (22) and intensive rehabilitation (23) align with emerging evidence linking reduced neuroinflammation and activity-dependent synaptic plasticity to improved neurological outcomes after SCI (24, 25). Notably, in patients ≥40 years, comorbidity burden—not motor score—became the dominant predictor, suggesting that age-related microvascular compromise magnifies the biological cost of systemic inflammation (26).

This work advances the field of thoracolumbar incomplete SCI prognostication through two distinct, impactful innovations. First, we are innovatively to quantify intramedullary T2-lesion length as a prognostic factor in thoracolumbar incomplete SCI at scale. Second, we integrated PLC integrity and decompression timing into a unified prediction model, quantifying their synergistic predictive effect. In all, The bedside-ready eight-factor model allows clinicians to estimate individual recovery probability with clinically meaningful precision (95% CI ± 5%). For a prototypical 35-year-old non-smoker with AIS C, intact PLC, 25 mm lesion and early surgery, predicted probability rises from 20 to 45%; conversely, delaying surgery beyond 24 h or extending the lesion to 50 mm halves this likelihood. These incremental probabilities can inform shared decision-making, triage to specialized centers and resource allocation. Economic modeling by Dvorak et al., based on a prospective Canadian cohort of 1,200 thoracolumbar SCI patients, indicates that protocolised early surgery (<24 h) combined with ≥3 h daily inpatient rehabilitation is associated with lifetime societal savings of approximately CAD $12,800 per patient (27). Notably, rehabilitation ≥3 h/day conferred significant benefit only when lesions were ≥30 mm or surgery was delayed, suggesting that enhanced therapy may partially compensate for unfavorable biology or late intervention. Rehabilitation ≥3 h/day only benefited those with lesions ≥30 mm or delayed surgery, indicating that resource allocation can be stratified by MRI lesion length.

Although consecutive enrolment limits selection bias, residual confounding due to insurance type and post-acute discharge destination remains possible. A key limitation of this study is the lack of external validation in an independent multicenter cohort. External validation is critical to confirm the model’s generalizability across different clinical settings, patient populations, and treatment protocols; future research should prioritize external validation of this model in an independent multicenter cohort, ideally including centers with diverse practice patterns (e.g., academic vs. community hospitals, different rehabilitation protocols). Furthermore, the lack of quantitative susceptibility mapping and diffusion tensor imaging—fractional anisotropy (DTI-FA) metrics may have led to an underestimation of axonal integrity. Unmeasured socioeconomic factors could also contribute to residual confounding. Advanced MRI measures, such as axial area, DTI-FA, and hemorrhage-to-edema ratio, were not available, and the composite AIS/SCIM outcome may overestimate actual walking independence. Future studies should incorporate more specific assessments, such as the Walking Index for Spinal Cord Injury II or 10-meter walk test (28). Lastly, relying on a single 12-month follow-up limits the ability to detect early plateaus or delayed recovery gains, highlighting the need for longitudinal cohorts with multiple time points to accurately characterize the entire recovery trajectory.

## 5 Conclusion

A concise eight-variable model accurately predicts 12-month functional recovery after thoracolumbar incomplete SCI and reveals context-dependent predictive weights. Implementation may enhance prognostic counseling and guide individualized treatment strategies.

## Data Availability

The original contributions presented in the study are included in the article/Supplementary material, further inquiries can be directed to the corresponding author.
