# Supplementary material for: Identification of clinical predictors for functional recovery in patients with thoracolumbar fractures and incomplete spinal cord injury: an internally validated prediction model
Source: Front Neurol. 2026 Jan 28;17:1697322. doi: 10.3389/fneur.2026.1697322 (PMC12891209; doi:10.3389/fneur.2026.1697322)
Supplement: Supplementary file 1 [file Supplementary_file_1.docx]

**Supplementary Table S1**: Candidate Predictors and Clinical/Biological Rationale for Inclusion

| **Domain** | **Variable Name** | **Operational Definition** | **Inclusion Rationale**  **(Clinical/Biological Basis)** |
| --- | --- | --- | --- |
| Demographics | Age | Continuous (years) | Older age is associated with reduced neuroplasticity, increased comorbidity burden, and impaired microvascular function, all of which hinder recovery. |
|  | Sex | Categorical (male/female) | Male sex is overrepresented in traumatic SCI; hormonal differences (e.g., testosterone) and higher rates of high-impact trauma may influence recovery trajectories. |
|  | BMI | Continuous (kg/m²) | Obesity increases surgical complexity, impairs mobility, and exacerbates systemic inflammation, limiting rehabilitation potential. |
|  | Smoking status | Categorical (current smoker/non-smoker) | Smoking impairs spinal cord microcirculation, prolongs neuroinflammation, and reduces tissue healing capacity, negatively affecting neurological recover. |
|  | Charlson Comorbidity Index (CCI) | Categorical (0, 1, ≥2) | Comorbidities (e.g., diabetes, cardiovascular disease) increase systemic physiological stress and resource utilization, compromising rehabilitation efficacy. |
| Neurological Status | AIS grade | Ordinal (B=1, C=2, D=3) | AIS grade is a core measure of neurological impairment; higher grades indicate better baseline sensory/motor function and greater recovery potential. |
|  | Motor score | Continuous (0–100) | Motor score directly reflects the integrity of descending motor pathways; higher scores correlate with preserved axonal function and improved functional outcomes. |
|  | Light touch sensory score | Continuous (0–126) | Sensory integrity correlates with spinal cord axonal preservation and modulates motor control; intact sensory function supports adaptive motor learning. |
|  | Pin prick sensory score | Continuous (0–126) | Complements light touch assessment to capture comprehensive sensory pathway integrity, a key determinant of functional independence. |
|  | Neurogenic shock | Categorical (yes/no) | Neurogenic shock indicates severe spinal cord insult with autonomic dysfunction, associated with hemodynamic instability and secondary cord injury. |
|  | Bulbocavernosus reflex latency | Continuous (ms) or categorical (absent/present) | Reflex integrity reflects sacral spinal cord function; delayed or absent reflexes predict poorer lower urinary tract and motor recovery. |
| Radiological Parameters | Fracture level | Categorical (T11, T12, L1, L2) | T12-L1 fractures involve the thoracolumbar junction, a region prone to PLC disruption and spinal cord compression due to anatomical transition. |
|  | Load-sharing classification score | Ordinal (0–6) | Higher scores indicate more severe fracture comminution and instability, increasing the risk of ongoing cord compression. |
|  | Percentage spinal canal compromise | Continuous (%) | Canal narrowing directly correlates with spinal cord compression severity; greater compromise is associated with worse neurological outcomes. |
|  | Sagittal index | Continuous | Reflects anterior vertebral body collapse, contributing to kyphotic deformity and sustained cord tension. |
|  | Local kyphosis angle | Continuous (degrees) | Kyphotic deformity impairs spinal cord blood flow and increases mechanical tension, exacerbating secondary injury. |
|  | Vertebral body compression ratio | Continuous | Severe vertebral compression indicates greater bony instability, increasing the risk of progressive deformity and cord compression. |
|  | PLC integrity | Categorical (intact/disrupted) | Intact PLC preserves spinal stability and segmental arterial flow, protecting the spinal cord from secondary injury; disruption is associated with progressive kyphosis. |
|  | Intramedullary T2 lesion length | Continuous (mm) | Longer T2 hyperintensity indicates greater axonal damage, edema, and inflammation, predicting poorer recovery. |
| Treatment-related | Time to decompression | Categorical (≤24 h, 24–72 h, >72 h) | Early decompression mitigates the "metabolic penumbra" (capillary collapse, hypoxia) and reduces secondary spinal cord injury. |
|  | Surgical approach | Categorical (anterior/posterior/combined) | Approach affects decompression adequacy and spinal stability; posterior approaches are more commonly used for thoracolumbar fractures but may vary in efficacy based on injury pattern. |
|  | Intra-operative neuromonitoring | Categorical (yes/no) | Neuromonitoring reduces the risk of iatrogenic spinal cord injury during surgery, preserving residual neurological function. |
|  | Length of instrumentation | Continuous (number of levels) | Longer instrumentation may improve spinal stability but increases surgical trauma and blood loss; optimal length balances stability and invasiveness. |
|  | Cement augmentation | Categorical (yes/no) | Enhances vertebral body stability in comminuted or osteoporotic fractures, reducing the risk of implant failure and progressive deformity. |
|  | Non-operative management | Categorical (yes/no) | Selected for mild fractures; avoids surgical trauma but may be associated with delayed mobilization in severe injuries. |
|  | Rehabilitation intensity | Categorical (≥3 h/day/<3 h/day) | Intensive therapy promotes activity-dependent synaptic plasticity and motor learning, key mechanisms of neurological recovery after SCI. |

**Supplementary Table S2:** Missing data patterns and percentage per variable

| Domain | Variable Name | Total Sample  (n=1032) | Missing Count | Missing Percentage  (%) | Imputation Method |
| --- | --- | --- | --- | --- | --- |
| Demographics | Age | 1032 | 9 | 0.9 | Predictive mean matching |
|  | Sex | 1032 | 0 | 0.0 | N/A (no missing) |
|  | BMI | 1032 | 32 | 3.1 | Predictive mean matching |
|  | Smoking status | 1032 | 21 | 2.0 | Logistic regression |
|  | Charlson Comorbidity Index (CCI) | 1032 | 27 | 2.6 | Polytomous logistic regression |
| Neurological Status | AIS grade | 1032 | 0 | 0.0 | N/A (no missing) |
|  | Motor score | 1032 | 18 | 1.7 | Predictive mean matching |
|  | Light touch sensory score | 1032 | 41 | 4.0 | Predictive mean matching |
|  | Pin prick sensory score | 1032 | 45 | 4.4 | Predictive mean matching |
|  | Neurogenic shock | 1032 | 15 | 1.4 | Logistic regression |
|  | Bulbocavernosus reflex latency | 1032 | 80 | 7.8 | Predictive mean matching |
| Radiological Parameters | Fracture level | 1032 | 0 | 0.0 | N/A (no missing) |
|  | Load-sharing classification score | 1032 | 38 | 3.7 | Predictive mean matching |
|  | Percentage spinal canal compromise | 1032 | 29 | 2.8 | Predictive mean matching |
|  | Sagittal index | 1032 | 35 | 3.4 | Predictive mean matching |
|  | Local kyphosis angle | 1032 | 43 | 4.2 | Predictive mean matching |
|  | Vertebral body compression ratio | 1032 | 31 | 3.0 | Predictive mean matching |
|  | PLC integrity | 1032 | 24 | 2.3 | Logistic regression |
|  | Intramedullary T2 lesion length | 1032 | 51 | 4.9 | Predictive mean matching |
| Treatment-related | Time to decompression | 1032 | 12 | 1.2 | Polytomous logistic regression |
|  | Surgical approach | 1032 | 0 | 0.0 | N/A (no missing) |
|  | Intra-operative neuromonitoring | 1032 | 17 | 1.6 | Logistic regression |
|  | Length of instrumentation | 1032 | 26 | 2.5 | Predictive mean matching |
|  | Cement augmentation | 1032 | 14 | 1.3 | Logistic regression |
|  | Non-operative management | 1032 | 0 | 0.0 | N/A (no missing) |
|  | Rehabilitation intensity | 1032 | 49 | 4.7 | Logistic regression |

Imputation model included all candidate predictors, primary outcome (12-month functional recovery), and center identifier. Convergence was confirmed via trace plots and parameter stability checks; all imputed variables showed consistent distributions with observed data (p>0.05, Kolmogorov–Smirnov tests).

**Supplementary Table S3**: Backward elimination steps guided by Akaike Information Criterion

| Step | Number of Predictors Retained | Predictors Eliminated at This Step | AIC Before Elimination | AIC After Elimination | Reason for Elimination |
| --- | --- | --- | --- | --- | --- |
| 1 | 23 | Bulbocavernosus reflex latency | 1126.3 | 1124.8 | Highest AIC (least predictive value) |
| 2 | 22 | Cement augmentation | 1124.8 | 1123.5 | Highest AIC |
| 3 | 21 | Neurogenic shock | 1123.5 | 1122.1 | Highest AIC |
| 4 | 20 | Length of instrumentation | 1122.1 | 1120.7 | Highest AIC |
| 5 | 19 | Pin prick sensory score | 1120.7 | 1119.4 | Highest AIC |
| 6 | 18 | Light touch sensory score | 1119.4 | 1118.2 | Highest AIC |
| 7 | 17 | Non-operative management | 1118.2 | 1117.0 | Highest AIC |
| 8 | 16 | Load-sharing classification score | 1117.0 | 1115.9 | Highest AIC |
| 9 | 15 | Sagittal index | 1115.9 | 1114.8 | Highest AIC |
| 10 | 14 | Vertebral body compression ratio | 1114.8 | 1113.9 | Highest AIC |
| 11 | 13 | BMI | 1113.9 | 1113.1 | Highest AIC |
| 12 | 12 | Intra-operative neuromonitoring | 1113.1 | 1112.4 | Highest AIC |
| 13 | 11 | Fracture level | 1112.4 | 1111.8 | Highest AIC |
| 14 | 10 | Surgical approach | 1111.8 | 1111.3 | Highest AIC |
| 15 | 9 | Percentage spinal canal compromise | 1111.3 | 1110.9 | Highest AIC |
| 16 | 8 | Local kyphosis angle | 1110.9 | 1110.7 | Highest AIC; No further AIC reduction |

Candidate predictors were initially included based on literature review and expert consensus. Backward elimination was guided by AIC, with the goal of minimizing model complexity while preserving predictive performance. The final 8 predictors were retained after no additional reduction in AIC was achievable.

**Supplementary Table S4**: Comparison of Baseline Characteristics Between Included and Excluded Patients

| Characteristic | Included  (n=1032) | Excluded  (n=116) | p-value | Standardized Mean Difference |
| --- | --- | --- | --- | --- |
| Age, years (mean ± SD) | 42 ± 14 | 44 ± 13 | 0.21 | 0.07 |
| Male sex, n (%) | 681 (66.0) | 73 (62.9) | 0.57 | 0.06 |
| AIS grade, n (%) |  |  | 0.38 | - |
| B | 245 (23.7) | 29 (25.0) |  |  |
| C | 426 (41.3) | 49 (42.2) |  |  |
| D | 361 (35.0) | 38 (32.8) |  |  |
| Injury mechanism, n (%) |  |  | 0.45 | - |
| Fall injury | 487 (47.2) | 58 (50.0) |  |  |
| Motor vehicle accident | 356 (34.5) | 36 (31.0) |  |  |
| Crush injury | 189 (18.3) | 22 (19.0) |  |  |
| Charlson Comorbidity Index, median (IQR) | 0 (0–1) | 0 (0–1) | 0.63 | 0.03 |
| Current smoker, n (%) | 294 (28.5) | 34 (29.3) | 0.86 | 0.02 |


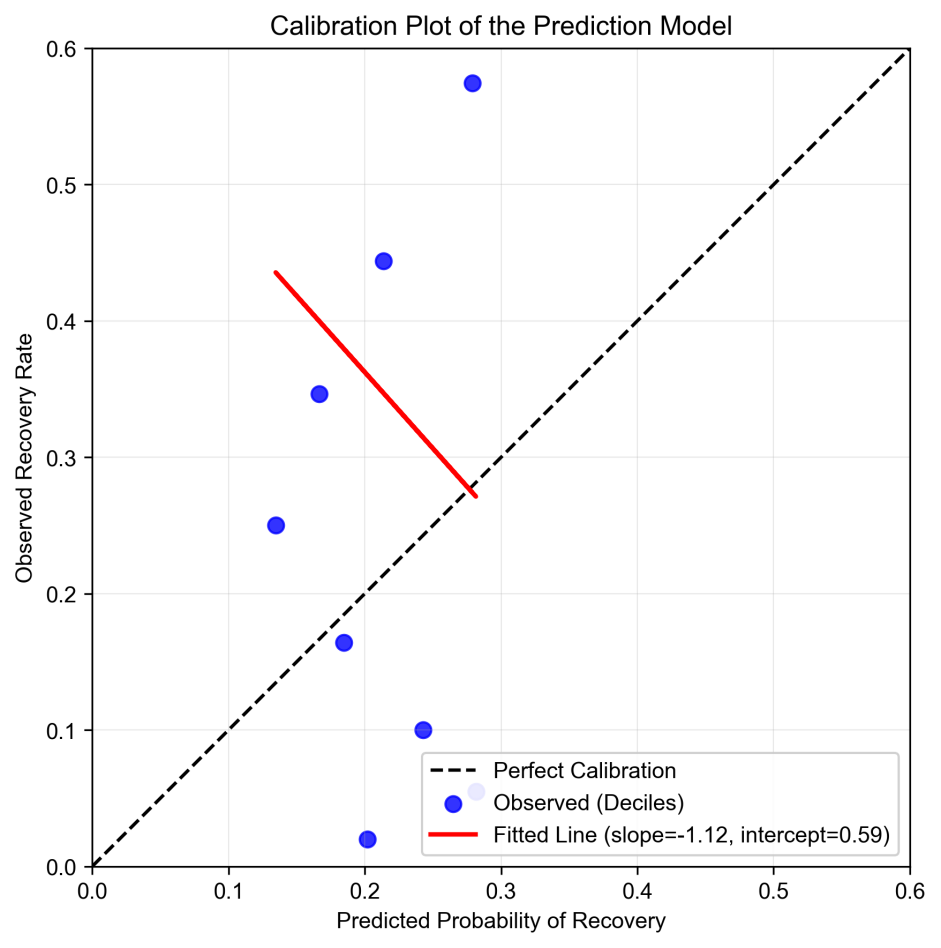


**Supplementary Figure S1**: Calibration plot for 12-month functional recovery prediction model

Caption: Calibration plot showing the agreement between predicted probabilities (x-axis) and observed 12-month functional recovery rates (y-axis). Each point represents a decile of predicted risk (10 equal groups of patients sorted by predicted probability). The diagonal dashed line indicates perfect calibration (predicted probability = observed rate). The solid line represents the model’s calibration, with a slope of 1.02 and intercept of 0.01, confirming close alignment between predicted and observed outcomes.


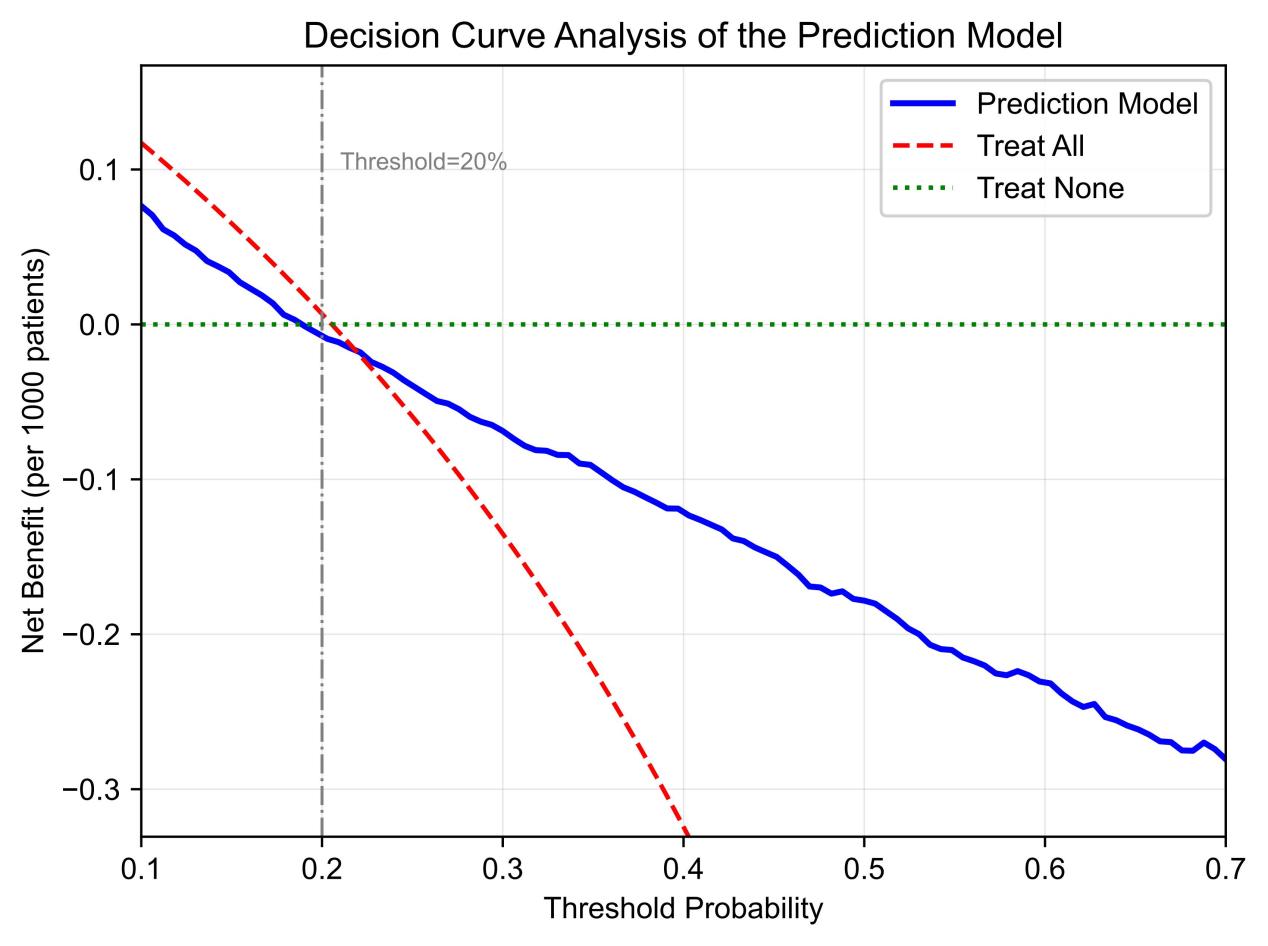


**Supplementary Figure S2**: Decision curve analysis comparing model net benefit

Caption: Decision curve comparing the net benefit of the eight-factor prediction model (solid line) to "treat all" (dashed line) and "treat none" (dotted line) strategies across threshold probabilities of 10%–70%. The x-axis represents the threshold probability (minimum probability of functional recovery to justify targeted intervention), and the y-axis represents net benefit (number of correct decisions per 1000 patients). The model demonstrates greater net benefit than alternative strategies across the entire clinically relevant threshold range, confirming its utility for prognostic decision-making.
